# The Art of Attracting Probationers

**Published:** 1919-09-20

**Authors:** 


### THE MATRONS' AND SISTERS' DEPARTMENT.

### THE ART OF ATTRACTING PROBATIONERS.

In the days when matrons had twenty times as many applicants as they wanted for every vacancy, the art of securing a good type of woman might be safely neglected. If half the number of candidates were preposterously unfitted to be nurses, there was still a good choice, and by process of selection sufficient reasonably satisfactory candidates could be decided on. All this vanished with the arrival of the war and the Women's Legions. New methods came into vogue. Attractive advertisements for young women covered the hoardings. Full-page announcements in every type of journal brought before the women of the country the attractions and importance of manifold kinds of work. And the hospitals which had never taken any trouble to secure workers found the stream running away from their doors. The position at the moment is that hospitals well before the public are getting plenty of applications, but are no longer embarrassed by the flocks who used to besiege their matron's offices. The average hospital finds itself only just able to keep the staff up to strength, and accepts many probationers who would not have had a chance in days gone by, while the institutions deficient in attractions are compelled to take almost anyone, and are perennially short-staffed.

It is tolerably clear that the old easy way of waiting until young women of the right type find out that the hospital needs probationers will not serve any longer. Matrons and managers must lay aside the haughty air of permitting, but not encouraging applications. They must go to meet the intending probationer, and they must cultivate the art of inducing the right kind of woman to desire before all things to become a hospital nurse. How is this to be done?

It is an open secret that the managers of the Metropolitan Asylums Board are greatly disappointed that the recent reforms in hours and salaries effected in that service have not led to a more striking increase in the number of applications for vacant posts. That it was the right policy to pursue and that it will result in raising the standard in everyone of their institutions and increasing their efficiency is indisputable. It has been carried through with far-sighted wisdom, and its influence will be widely felt outside the particular services involved. But it is significant that at present it has failed to appeal to the people from whom probationers are drawn. This is no doubt partly owing to the fact that the young women who would be welcomed have not yet come to know of the improvements which have taken place in their interests. More intelligent publicity is required. But it also indicates that material advantages have less sway over the minds of candidates than was popularly believed.

It is impossible to prescribe hard-and-fast methods for securing probationers, because circumstances differ, and the tactics to observe, say, in a university city, are different from those which are needed in a manufacturing town or a country locality. But whatever class of worker is required, whatever the conditions under which she may be called to labour, there is one guide to be observed in attempting to draw women into the nursing service, and that is to place before them not so much what they will get as what they can give. For very shame let the conditions of service be such as compare well with other forms of occupation. But that will not prove the real lever. Deep in the heart of every woman worth her salt, is the desire to make something worth having of her life. The brave instincts of adolescence all point to self-dedication, to heroic deeds, to achievement. Neglected or ridiculed such aspirations perish, or become diverted into the track of some cheap love affair. They are too often ignored in casting the net for candidates. Put before the girl who cherishes these secret aspirations towards a life higher than she has yet attained the purposeful and dignified ideals of the nurse, and she will react instantaneously to the vocation. She cares little in reality for assurances that her training will be easier, her salary more, in one place than in another. Her impulse is to do and dare. And it will take a good deal to discourage her when once she has started.

Thus the aim of the hospitals and infirmaries who want good probationers should be to get into touch with the women who feel the need for having a purpose in life, and to bring to their notice various aspects of the nurse's life likely to appeal to them. There are many ways, both public and private, of doing this. By reason of a defective psychology managers are allowing all the best candidates to slip through their fingers.

